# ‘And don't say everything will be normal!’: An international cross‐sectional survey on the patients’ unmet sexual wellbeing needs after ostomy formation

**DOI:** 10.1111/codi.70264

**Published:** 2025-10-06

**Authors:** Simona Fourie, Jan Bornschein, Christine Norton, Wladyslawa Czuber‐Dochan

**Affiliations:** ^1^ Radcliffe Department of Medicine University of Oxford Oxford UK; ^2^ Florence Nightingale Faculty of Nursing, Midwifery and Palliative Care King's College London London UK

**Keywords:** sexual wellbeing, stoma formation, survey

## Abstract

**Background:**

Stoma formation for inflammatory bowel disease, cancer or trauma can adversely affect sexual function and overall sexual wellbeing. There is a recognised unmet need to address patients' concerns within clinical practice. This study aimed to identify patient‐reported sexual wellbeing concerns, explore experiences of discussing these issues within clinical settings and determine patients' priorities regarding their sexual wellbeing needs.

**Methods:**

An international, web‐based cross‐sectional survey was disseminated via social media across English‐speaking countries, in collaboration with local ostomy and inflammatory bowel disease charities.

**Results:**

A total of 370 participants completed the anonymous survey between March 2023 and March 2024. Over 61% reported ongoing concerns related to sexual wellbeing, while 68% had not received any information regarding the potential impact of a stoma on their sexual life. Despite this, 89% expressed a desire for such information. When information was provided, surgeons were the most frequently reported source.

Primary concerns identified were body image (32%), appliance‐related issues (29.7%), difficulties in intimate relationships (28.1%) and reduced self‐confidence (9.9%). Over half of respondents preferred that all health professionals address sexual wellbeing as a routine part of care. Participants also recommended a variety of information resources. Age and time since stoma formation were significantly associated with the extent of concerns (*p* < 0.05).

**Conclusions:**

Sexual wellbeing concerns following stoma formation are common and frequently unmet. The lack of information provision contributes to patient distress. The routine integration of sexual wellbeing discussions, supported by accessible and diverse resources, is essential to delivering holistic, patient‐centred stoma care.

**What does this paper add to the literature?**

This study highlights sexual wellbeing as a significant and often unmet need among individuals living with a permanent stoma, with over 61% reporting concerns and 89% expressing a desire for more information. Key issues identified include body image, appliance‐related challenges and difficulties in intimate relationships. The findings emphasise the need for multifactorial, patient‐centred interventions to address this critical gap in support and improve holistic stoma care.

## INTRODUCTION

Stoma formation, undertaken as surgical treatment for conditions such as inflammatory bowel disease (IBD), cancer or trauma, significantly affects sexual wellbeing (SWB). SWB is a multidimensional concept encompassing bio‐psycho‐social aspects of sexuality and has been defined as ‘*sexual emotions and cognitions which include feeling safe, respected, comfortable, confident, autonomous, secure, and able to work through change, challenges, and past traumas”* [1]. Changes in SWB, whether manifesting as sexual dysfunction or adjustments in intimate relationships, represent key psychosocial concerns for individuals living with a stoma [2, 3, 4]. Body image, social functioning, sexual function and self‐esteem are all commonly affected following stoma formation [5, 6, 7].

Previous studies have highlighted a clear unmet need from patients' perspective to initiate discussions about the potential impact of surgery on sexual function [8, 9]. Individuals with IBD and cancer survivors frequently report SWB‐related concerns yet often receive insufficient support in clinical care settings [3, 4, 10, 11, 12, 13]. Sexual difficulties are known to negatively influence overall quality of life (QoL) among ostomates [14, 15, 16], with one study reporting that up to 80% of men experienced sexual problems 2 years after colostomy formation [17]. Despite well‐documented post‐operative issues such as erectile dysfunction or dyspareunia, 80–85% of patients report not receiving preoperative information regarding these potential outcomes, with potential implications for consent to procedures [9].

When unaddressed, these concerns may exacerbate psycho‐emotional distress. For example, 42% of individuals with Crohn's disease and an ostomy [18] and 37% of those with colorectal cancer [19, 20] report ongoing distress related to SWB. Given the increasing recognition of the need for a holistic approach to post‐stoma care and QoL improvement [21], concerns related to intimacy and sexuality must not be overlooked, particularly considering unmet needs regarding patients' SWB concerns [22, 23].

This study aimed to address a critical gap by: a) identifying patient‐reported concerns about SWB following stoma formation; b) exploring experiences of discussing these issues in clinical care and c) establishing patient‐defined priorities in relation to SWB support.

## METHODS

### Study design

This international cross‐sectional survey formed part of a mixed‐methods exploratory sequential study [24] and is reported in accordance with the Checklist for Reporting Results of Internet E‐Surveys (CHERRIES) [25].

### Participant recruitment and selection

Data were collected via a voluntary, self‐administered online questionnaire between March 2023 and March 2024. The study was advertised through social media platforms and in collaboration with relevant charities and patient support groups across English‐speaking regions, including the United Kingdom, United States of America, Canada, Australia and New Zealand. A target sample size of 350 participants was set to balance statistical power and feasibility. Ethical approval was obtained from the University of Oxford (Reference: R82054/RE001).

### Survey design

The survey was co‐developed by the research team in collaboration with patient representatives and refined following feedback from five individuals with lived experience of a stoma. It focused on four main domains: (1) demographic characteristics, (2) presence of SWB concerns, (3) experiences of receiving SWB‐related information and (4) patient‐informed suggestions for future clinical practice.

The survey comprised 25 questions (multiple‐choice or free text), of which 23 questions were mandatory. Optional questions aimed to capture supplementary free‐text responses linked to the preceding multiple‐choice items, or to accommodate content not applicable to all respondents. Participants were also asked to rate their level of concern and their expectation to discuss SWB on a scale from 1 to 10 (where 1 indicated *no concern or expectation* and 10 represented the *highest level of concern or expectation*). The survey was hosted on an online platform. Once submitted, responses could not be revised. Incomplete questionnaires could not be submitted. Informed consent was obtained through a mandatory tick box prior to participation.

### Analysis

Descriptive statistics were used to summarise quantitative data. Categorical variables were described and analysed for correlations by using Chi‐square or Fisher's exact test, as applicable. Non‐parametric tests (Kruskal–Wallis or Mann–Whitney *U*) were used for comparisons of numeric variables. Pearson's correlation coefficient was calculated to examine relationships between continuous variables. Statistical significance was set at *p* ≤ 0.05, with Bonferroni correction applied for multiple comparisons. Data analyses were conducted using IBM SPSS Statistics, version 29.0.

Qualitative data from free‐text responses were analysed using content analysis [26], with thematic categorisation of emerging findings. Verbatim quotes (italicised) are presented to illustrate key themes, accompanied by relevant participant characteristics. Square brackets […] indicate that a quote has been slightly modified to aid readability.

## RESULTS

A total of 370 participants completed the survey (*F* = 219, *M* = 151). The median age was 42.3 years (range 18–85 years), and the median time since stoma formation was 3 years (range 2 weeks to 55 years). Further participant characteristics are presented in Table 1. Although the country of residence was not collected within the survey, 97 participants who consented to subsequent interviews provided information about their geographical location.

**TABLE 1:** Study population characteristics.

	Category	*N* (%)
Sex	Female	219 (59.1%)
	Male	151 (40.9%)
Marital status	Married/in a relationship	218 (58.9%)
	Single	115 (31.0%)
	Divorced/separated	28 (7.5%)
	Widowed	9 (2.4%)
Ethnicity	White	285 (77%)
	Asian	35 (9.4%)
	Black	9 (2.4%)
	Mixed background	33 (8.9%)
	Other	8 (2.2%)
Indication for stoma	Cancer	77 (20.8%)
	IBD	209 (56.4%)
	Trauma	23 (6.2%)
	Other	61 (16.5%)
Stoma type	Colostomy	133 (35.9%)
	Ileostomy	223 (60.2%)
	Urostomy	14 (3.7%)
Rectal stump	Yes	152 (41%)
	No	218 (58.9%)
Parastomal hernia	Yes	99 (26.7%)
	No	271 (73.2%)
Active cancer treatment^a^	Yes	25 (6.77%)

^a^ For those who had stoma as a result of cancer.

### Presence of sexual wellbeing concerns

Participants were asked to retrospectively rank their recalled level of concern regarding SWB at the time of stoma formation. Of the 38.9% (*n* = 121) who responded to this optional question, the mean self‐reported SWB concern score was 5.7 out of 10. Current concerns related to SWB were reported by 61% of participants (*n* = 252); this question was also optional and may not have been applicable to all respondents. Several statistically significant differences between stoma types are presented in Table 2.

**TABLE 2:** Survey responses/type of stoma and topics of the survey (percentage within the stoma group).

	Response	Colostomy	Ileostomy	Urostomy	Total	*p*‐Value
Current SWB concern	Yes	69 (51.9%)	152 (68%)	5 (35.7%)	226 (61.1%)	0.002
	No	64 (48.1%)	71 (32%)	9 (64.3%)	144 (38.9%)	
Advice received	Yes	53 (39.7%)	77 (34.7%)	10 (71.4%)	141 (38.1%)	0.021
	No	80 (60.3%)	145 (65.3%)	4 (28.6%)	229 (61.9%)	
Who initiated SWB discussion	Gastro	0 (0%)	4 (1.8%)	0 (0%)	4 (2.5%)	<0.0001
	Nurse	10 (7.5%)	28 (12.6%)	0 (0%)	38 (24.1%)	
	Surgeon	36 (27.1%)	32 (14.14%)	9 (64.3%)	77 (48.7%)	
	Patient	11 (3%)	25 (6.8%)	3 (0.8%)	39 (24.7%)	
Who should initiate discussion	GP	1 (0.8%)	1 (0.5%)	0 (0%)	2 (0.5%)	0.005
	Nurse Specialist	25 (18.8%)	37 (16.7%)	2 (14.3%)	64 (17.3)	
	Stoma Nurse	23 (18.8%)	41 (18.5%)	0 (0%)	64 (17.3%)	
	Surgeon	23 (17.3%)	14 (6.3%)	5 (35.7%)	42 (11.4%)	
	None	4 (3.0%)	2 (0.9%)	0 (0%)	6 (1.6%)	
	All of the above	54 (42.9%)	131 (35.4%)	7 (50%)	192 (51.9%)	
Want the partner present	Yes	67 (50.4%)	97 (43.7%)	9 (64.3%)	173 (46.8%)	0.501
	No	41 (30.8%)	80 (36%)	4 (28.6%)	125 (33.8%)	
	Other	25 (18.8%)	46 (43.7%)	1 (7.1%)	72 (19.4%)	
Preferred time of discussion	Before surgery	37 (27.8%)	59 (26.6%)	8 (57.1%)	104 (28.1%)	0.008
	After surgery	18 (13.5%)		1 (7.1%)	31 (8.4%)	
	At any point of care	78 (58.6%)	12 (5.4%)	5 (35.7%)	235 (63.2%)	
			152 (68%)			
Suggested sources of information	Leaflet	29 (21.8%)	17 (7.7%)	8 (57.1%)	54 (14.6%)	<0.001
	Website	31 (23.3%)	67 (30.2%)	1 (7.1%)	99 (26.8%)	
	Educational Events	8 (2.1%)	18 (4.8%)	0 (0%)	26 (6.7%)	
	Other (peer support)	3 (0.8%)	4 (1%)	0 (0%)	8 (2.2%)	
	All of them	63 (47.7%)	116 (52.3%)	5 (35.7%)	184 (49.7%)	

Free‐text responses provided further insight into the SWB concerns following stoma formation in some participants:

No consideration is given to how stressful it is to have such a major change in the way your body functions. I don't see my stoma as a problem, it saved my life. I wanted help to figure out how to adapt to a new way of living, sex included. (F, 48, IBD, Ileostomy)

Participants with IBD reported the highest mean score for SWB concerns at the time of stoma formation (8/10), compared to a mean score of 3/10 among respondents with other diagnoses (*p* < 0.001). Among ethnic groups, individuals from Asian and mixed backgrounds expressed the highest levels of concern, also reporting a mean score of 8/10 (*p* = 0.002). Free‐text responses provided additional context for these elevated concerns among participants with IBD:

Crohn's alone made my intimate life difficult; [the] stoma just added an extra layer to that. (F, 32, IBD, ileostomy)

Content analysis identified 254 discrete concerns, categorised into four key domains: body image, appliance (bag)‐related issues, intimate relationships and self‐confidence (Table 3). Several participants reported more than one concern, accounting for the difference between the number of participants who reported concerns (*n* = 226) and the total number of concerns identified (*n* = 254).

**TABLE 3:** Concern domains with supporting quotes from free‐text responses.

Domain of concern	Quote
Body image (scars, look of the stoma, parastomal hernia, feeling unattractive)	*Every time our bodies change, our feelings about them and about intimacy can change. (F, 41, IBD, colostomy)*
*n* = 83 (32.6%)	*I hate my body (F, 59, cancer, colostomy)*
Appliance‐related concerns (functioning of the bag, leakage and fear of leakage, noises, odour) *n* = 75 (29.5%)	*My partner wanted spontaneity, but my bag made the experience more mechanical for her (M, 30, IBD, ileostomy)*
Intimate relationships (fear of rejection, maintaining existing relationships, prospects of dating, sexual dysfunction)	*The loss of my intimate relationship with my husband has deeply saddened us both. We have never had difficulties before, and this has come as a shock. I have been physically impacted, the loss of my rectum has made it painful, sore and fearful of intimacy, and this has caused mental apprehension and resistance to being intimate with my partner. It has caused a loss in our relationship that wasn't anticipated, and we don't know how to overcome it. (F, 54, cancer, colostomy)*
*n* = 71 (27.9%)	*When you're told how to change your bag is little talk about how that bag changes your life. I know I had issues in my marriage, but the bag ended it. I am still grateful I am alive. (F, 54, trauma, colostomy)*
Self‐confidence (low self‐esteem, loss of confidence in own ability to engage in sexual relationships) *n* = 25 (9.8%)	*I was worried about having to talk about my Crohn's to people I would date. Now is much worse, stoma noises can be very upsetting and have to bring this up very early on. Still not got the guts to date (F, 18, IBD, colostomy)*

Abbreviation: *n*, number of concerns.

### Experiences of receiving information pertaining to sexual wellbeing

Almost two‐thirds of participants (61.8%) reported that SWB had not been discussed with healthcare professionals (HCPs):

The subject of sexual activity or intimacy has never arisen, therefore I have never brought it up myself through fear that it is not wanted to be spoken about. (F, 44, IBD, ileostomy)

Despite the absence of such discussions, 89% of participants expressed a desire for SWB to be routinely addressed, recognising its relevance and importance:

After a traumatic event that has a life‐changing effect, you would expect that relationships and intimacy would be considered important. Nobody had ever mentioned it! (F, 47, trauma, colostomy)

Table 4 presents the sources of SWB‐related information. Some participants recounted dismissive responses or a lack of meaningful explanation when concerns were raised:

As a young adult at the time, there should have been consideration about my future. I asked about dating with a bag. It was laughed off, and I was told I had more important things to worry about at that time. [I] Never asked again! (F, 28, Hirschsprung's disease, colostomy)

In emergency surgery this topic needs to be brought up several weeks after operation. Using the aid of products, [such as] a belt to help in intimate moments. [HCPs should] make it is clear is okay to talk about this. And don‘t say everything will be normal, it is not setting realistic expectations. (F, 54, trauma, colostomy).

Participants were also asked whether they would prefer their partner to be present during SWB‐related discussions with HCPs. Nearly half (48.1%) indicated they would welcome this. However, 19% selected ‘*Other’* and elaborated that patients should always be consulted beforehand, as some individuals may be in difficult or unsafe relationships, in which case such discussions could cause distress or pose a risk of harm.

**TABLE 4:** Information/advice received in different diagnostic groups.

SWB advice received	Cancer	IBD	Trauma	Other	*p*‐Value
Yes	41 (53.2%)^a^	75 (36.1%)	8 (34.8%)	17 (26.2%)	0.009
No	36 (46.8%)	133 (63.9%)	15 (65.2%)	45 (73.8%)	
*Who discussed SWB*					
Gastroenterologist	0 (0%)	4 (1.9%)	0 (0%)	0 (0%)	<0.001
Nurse	4 (5.1%)	28 (13.3%)	3 (13.1%)	3 (4.9%)	
Surgeon	34 (4.1%)	29 (13.9%)	11 (47.9%)	3 (4.9%)	

^a^ Percentages within the diagnostic group.

### Suggestions for future practice

Participants provided numerous recommendations on how SWB should be addressed within clinical practice. These included clear preferences regarding which HCPs should raise the topic and the optimal timing in relation to stoma formation. There was a strong consensus that all HCPs involved in a patient's care should be equipped to engage in discussions about SWB. Participants also expressed resources, favouring an ‘all options’ approach that included printed leaflets, dedicated websites and educational sessions.

Among free‐text responses, peer support, particularly opportunities to meet or connect with other ostomates was the most frequently suggested additional source of support. Participants further emphasised the importance of realistic and inclusive information tailored to a range of identities and life stages:

No LGBTQ info [was] available on this [SWB]. Stoma leaflets pictured 70+ [person] on front page. [This] suggested [that] only older people may have intimacy issues. (F, 26, IBD, ileostomy)

A near‐unanimous view was that HCPs should initiate conversations about SWB, even when they may not have immediate answers, to acknowledge and validate these concerns. Participants highlighted the need for improved awareness of the broader psychosocial impact of stoma formation, beyond the practical management of the stoma itself:

I think that all people involved in one's healthcare should be prepared to have honest conversations about sexual‐related concerns that people may have. I don‘t expect expert advice but at least signposting or recognition of issues that require expert review and referral. (M, 59, cancer, colostomy)

A summary of the integrated results and participant suggestions is provided in Table 5.

**TABLE 5:** Summary of findings with data integration.

Theme	Quantitative data	Qualitative data	Meta inferences
Presence of sexual wellbeing concerns	38.9% had SWB concerns pre stoma formation 61% had SWB concerns post‐stoma formation	*I asked and found the topic [sexual wellbeing] was avoided and not gone into any detail. I was worried and concerned*.	The increase in SWB concerns post‐stoma formation (from 38.9% to 61%) suggests that undergoing the procedure amplifies anxieties about intimacy and relationships. The qualitative data reinforces this, showing that patients feel SWB is often ignored or not discussed adequately, leading to increased distress.
Experiences of receiving information pertaining to sexual wellbeing	61.8% have not discussed SWB with HCPs 89% wanted to discuss	*After a traumatic event that has life changing effect you would expect that relationships and intimacy would be considered important. Nobody had ever mentioned it!*	A significant gap exists between patients' need for SWB discussions (89%) and actual discussions (61.8% reported no discussion). Patients express frustration over the absence of SWB discussions, highlighting a perceived neglect of this important aspect of post‐surgical recovery and wellbeing.
Suggestions for future practice	63.2% wanted SWB to be discussed at any point of care 53% wanted information to be given in a variety of forms	*I think that all people involved in one's health care should be prepared to have honest conversations about sexual related concerns that people may have. I don‘t expect expert advice, but at least signposting or recognise issues that require expert review and referral*.	The data indicates strong support (63.2%) for integrating SWB discussions into all stages of care, reinforcing the need for a proactive approach. A preference for diverse informational formats (53%) suggests that healthcare providers should adopt multimodal communication strategies, including verbal discussions, written materials and digital resources. The qualitative responses stress the importance of healthcare professionals being open to SWB conversations, even if they lack expertise, to ensure appropriate signposting and referrals.

### Correlations

Analysis of associations between numerical variables revealed several significant correlations between age, duration of stoma, initial level of concern, expectation to discuss SWB and reported current concerns (Table 6). Specifically, older participants and those with a longer duration since stoma formation were less likely to report concerns (median age of those with and those without concerns: 38 vs. 46 years [range 18–85]; *p* < 0.001; median duration after stoma formation of those with and those without concerns: 2.5 vs. 4 years [range 0.02–55]; *p* = 0.002). Additionally, a higher initial level of concern was associated with a greater expectation to discuss SWB.

**TABLE 6:** Pearson correlation between numerical items.

		Age	Stoma duration	Concerns pre stoma	Expectation to discuss	Answered concerns
Age	Pearson correlation	1	0.137**	−0.207**	−0.080	0.161**
	Sig. (2 tailed)		0.008	0.001	0.124	0.002
	*N*	370	370	370	370	370
Stoma duration	Pearson correlation	0.137**	1	0.017	−0.026	−0.38
	Sig. (2 tailed)	0.008		0.742	0.623	0.471
	*N*	370	370	370	370	370
Concerns pre stoma	Pearson correlation	−0.207**	0.017	1	0.289**	0.023
	Sig. (2 tailed)	<0.001	0.742		0.001	0.655
	*N*	370	370	370	370	370
Expectation to discuss SWB	Pearson correlation	−0.080	−0.026	0.289**	1	0.001
	Sig. (2 tailed)	0.124	0.623	0.001		0.978
	*N*	370	370	370	370	370
Answered SWB concerns	Pearson correlation	0.161**	−0.038	0.023	0.001	1
	Sig. (2 tailed)	0.002	0.471	0.655	0.978	
	*N*	370	370	370	370	370

*Note*: Age is reported in years; stoma duration is reported in months. Scores for concerns, expectations to discuss sexual well‐being (SWB) and level of answered concerns are presented on a 1–10 scale, where 1 indicates *no concern*, *no expectation to discuss SWB*, or *no concerns addressed*, and 10 indicates *highest concern*, *strongest expectation to discuss SWB*, or *all concerns addressed*.

** Correlation is significant at the 0.01 level (2‐tailed).

## DISCUSSION

To our knowledge, this is the first cross‐sectional survey specifically exploring SWB concerns from the patient's perspective following stoma formation. Despite the recognised importance of SWB, it remains often overlooked in routine care, with approximately two‐thirds of participants reporting a lack of information on the subject. The principal concerns identified in this study related to four interrelated domains: body image, appliance‐related concerns, the impact on intimate relationships and self‐confidence. Although 89% of participants expressed a desire for SWB to be addressed routinely, only 37.8% reported having discussed the topic with HCPs. Previous studies similarly highlight that SWB is frequently omitted from clinical conversations [8, 13], despite being a priority for patients [13, 26]. Our findings highlight the mismatch between patient‐reported needs and the responses of HCPs, a gap also evident in a recent patient‐led research on colorectal and pelvic floor surgery [8]. This disconnect represents an unmet need in post‐stoma care and highlights the limitations of a purely biomedical approach that fails to account for the psychosocial ramifications of life with a stoma.

Adjusting to a stoma is inherently complex, and the challenge is compounded when psychological and relational needs are not recognised or addressed. Addressing this gap is essential to provide holistic, patient‐centred care and improving QoL [27]. Previous research suggests that some surgeons do not perceive reduced QoL as being directly attributed to the stoma itself [28], potentially limiting the support offered and impending adaptation.

The four domains of concern identified in this study are consistent with previous literature [29]. Body image and appliance‐related issues, such as concerns about leakage, have been previously linked to impaired social functioning among individuals with IBD [5]. Similarly, reduced self‐esteem and emotional distress have been shown to negatively affect sexual QoL for IBD populations [30, 31]. Notably, a previous survey of 540 ostomates found that 54% reported low body confidence [8], aligning with our findings.

Our data demonstrate that body image was the most frequently reported concern, irrespective of age, gender and marital status. This is consistent with prior research indicating the negative impact of stoma surgery on both body image and sexual function [6, 21, 30]. Altered body image has been associated with increased psychological distress, particularly in younger individuals with IBD [32, 33]. In our study, this was corroborated by the highest levels of SWB concerns reported among younger participants, especially those with IBD. While many IBD participants described their stoma as life‐enhancing, concerns regarding SWB remained significant. Findings from previous research have documented the adverse effects of impaired body image on QoL and psychological wellbeing following stoma formation [34, 35, 36], all of which are likely factors contributing to increased SWB concerns post‐surgery. This echoes findings that although stoma formation may improve overall health‐related QoL in severe Crohn's disease [5, 37], psychological support is still required to address distress and adjustment challenges.

Leakage and fear of leakage emerged as one of the most frequently reported concerns, highlighting their substantial impact on SWB. Both leakage and odour are well‐documented contributors to stigma [38], with leakage affecting up to 75% of ostomates [39]. Given its frequency and emotional burden, targeted clinical strategies to address leakage‐related anxiety may meaningfully enhance patients' SWB.

Participants' difficulties in accessing information on SWB are consistent with existing evidence showing that timely information can facilitate adjustment after stoma surgery. Multidisciplinary approaches are crucial concerns [13, 40, 41] recognising the distinct yet complementary roles of various HCPs in addressing complex QoL issues. Notably, 87% of HCPs have reported lacking appropriate tools to discuss sexuality with patients [13], highlighting a need for better training and resources. Equipping HCPs to support patients in navigating post‐surgery challenges, including establishing realistic expectations for recovery, should be a priority [42].

A recent patient‐led survey reported that 79% of participants received no advice on SWB postoperatively [8], mirroring our findings. Many participants emphasised that information should not be confined to the perioperative period but should be delivered at multiple stages along the care pathway. This is supported by research showing that only 25% of patients with bowel cancer recalled dietary advice post‐treatment [43], indicating the need for repeated, tailored communication over time.

Barriers to delivering SWB education include HCPs' limited knowledge, discomfort with the topic, personal beliefs, time constraints and perceptions that sexuality is not a clinical priority, factors identified in a recent systematic review [26]. In addition, SWB information must be relevant and responsive to patients' lived experiences, incorporating consideration of social, relational, and cultural contexts [44], including the influence of cultural, social and relational contexts.

Our study also identified significant gaps in culturally sensitive care and inclusivity. Participants from ethnic minority backgrounds and sexual minority groups described a lack of tailored information, reinforcing previously reported concerns. Elias [45] and colleagues have proposed practical recommendations for delivering inclusive, sensitive care for sexual and gender minorities living with IBD. These include personalised approaches that acknowledge specific SWB needs, such as considerations for anoreceptive intercourse and gender‐affirming care and advocate for multidisciplinary teams to possess adequate knowledge of the challenges faced by these populations.

### Strengths and limitations

This study has several limitations. First, selection bias may have arisen from recruitment via IBD‐specific platforms and the inclusion of only English‐speaking participants. As a result, the findings may not be generalisable to those from diverse cultural, linguistic or geographic backgrounds, who may experience the impact of stoma formation on SWB differently. Additionally, self‐selection bias may have skewed the sample towards individuals with a particular interest in, or concern about, SWB.

Despite these limitations, the study has notable strengths. Its web‐based, international design enabled participation from individuals across a range of healthcare settings, including both public and private systems. This facilitated the inclusion of a broad range of experiences and perspectives, thereby enhancing the relevance and applicability of the findings. The large sample size and patient‐centred focus further strengthen the study's contribution to understanding the psychosocial and sexual impact of stoma formation.

## CONCLUSION

SWB concerns frequently arise following stoma formation, even among individuals who initially reported no such concerns. As no single demographic or clinical factor was found to be significantly predictive of SWB concerns, we recommend that SWB concerns be addressed universally, irrespective of stoma type, age, gender, sexual activity, sexual orientation or marital status.

To improve patient support and communication, HCPs should adopt a proactive approach in initiating discussions about SWB, provide timely and individualised information and offer access to a range of inclusive educational resources. The significant differences observed in patient experiences highlight the need for a tailored, inclusive and patient‐centred approach to care. Further work should focus on developing validated assessment tools to support the identification of individuals with significant SWB concerns. Additionally, the design of targeted educational interventions could help reduce the burden on HCPs while addressing patients' SWB needs in the context of life after stoma formation.

## AUTHOR CONTRIBUTIONS

**Simona Fourie:** Conceptualization; investigation; funding acquisition; writing – original draft; methodology; formal analysis; project administration. **Jan Bornschein:** Methodology; writing – review and editing; software; data curation; visualization. **Christine Norton:** Writing – review and editing; supervision; methodology; visualization. **Wladyslawa Czuber‐Dochan:** Visualization; writing – review and editing; supervision; methodology.

## FUNDING INFORMATION

Investigator‐led research grant Convatec was awarded to SF to conduct this study.

## CONFLICT OF INTEREST STATEMENT

SF received speaker fees from Convatec and Janssen. JB received advisory fees from Flynn Pharma Ltd., UK. CN declares speaker fees from: Medscape, Merck Pharmaceutical; Tillotts Pharma UK, Lilly. Pfizer advisory board. WCD declares speaker fees from Dr Falk Pharma and Pharmacosmos.

## ETHICS STATEMENT

Ethical approval was obtained from the University of Oxford (Reference: R82054/RE001).

## Data Availability

The data that support the findings of this study are available on request from the corresponding author. The data are not publicly available due to privacy or ethical restrictions.
